# Cynidae Pigmentation

**DOI:** 10.4269/ajtmh.21-0381

**Published:** 2021-09-27

**Authors:** M. Prarthana, Karthikeyan Kaliaperumal

**Affiliations:** Department of Dermatology, Venereology and Leprosy, Sri Manakula Vinayakar Medical College and Hospital, Puducherry, India

## CASE CHARACTERISTICS

**Figure 1.**
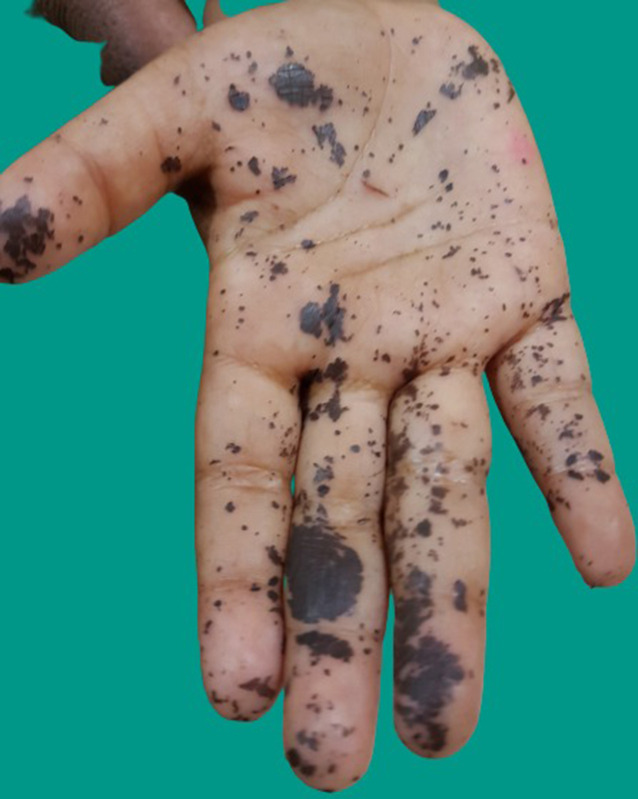
Black patchy macules of varying size and shape as ink splatter appearance. This figure appears in color at www.ajtmh.org.

A 5–year-old boy presented with a history of sudden onset of asymptomatic dark patchy pigmentation over his right hand for a duration of 2 days which could not be washed off with soap and water. The child gave history of playing in the fields along with his friends catching insects and butterflies. The parents explained the presence of similar kind of lesion among his play mates. On examination, right palm showed black patchy pigmentation as discrete macules of varying sizes as ink splatter appearance which to spread along the dermatoglyphics (Figure 1). Diagnosis of cynidae pigmentation was made based on the history, tropical nature of the occurrence of the disease, and clinical examination, and the parents were reassured regarding treatment.

## DISCUSSION

Cutaneous pigmentation because of cynidae bugs are mostly underreported may either be due to its transient nature or self-awareness of its innocuousness by the people belonging to the indigenous locality. The occurrence of burrow bugs (*Chilocoris assmuthi*) has been reported in various parts of India. Cynidae fall under the superfamily pentatomoideae with a common name of “burrowing bugs” because of its natural habitat of staying underground, feeding on roots of plants.^1^ These insects have a dark ovoid body with a flattened head and spiny legs with modified tarsi and tibia adapted to its habit of digging.^2^ The pigmentation is because of the secretion of a stinky hydrocarbonate produced by special glands located in the thorax in adult bugs and abdomen in nymph in response to stress as in defense and are also said to have antimicrobial and chemoattractant property.^3^^,^^4^ Pigmentation that occurs is because of crushing of the insects and a blackish brown tint develops at the contact site almost immediately and persists for around 10–15 days. Dermascopy reveals cluster of numerous bizarre-shaped shiny brown globules, clods, and granules with a superficial stuck on appearance.^5^ The condition undergoes spontaneous resolution. Differential diagnosis includes Peutz–Jeghers syndrome, Laugier–Hunziker syndrome, junctional melanocytic naevi, acral lentigines, resolving petechieae, and tinea nigra.

Asymptomatic exogenous pigmentation may simulate many dermatologic ailments but a careful history and examination points us toward the cause. The tip off in this case would be prevalence of the bug in India, definite history of outdoor activity before the onset of lesions and its characteristic pigmentation.
